# Self-reporting quality of life in mild-to-moderate Alzheimer’s disease and Lewy body dementia: Comparing capability and health-focused measures using response process validation

**DOI:** 10.1016/j.socscimed.2026.119102

**Published:** 2026-02-18

**Authors:** Irina Kinchin, Jayne Conlon, Erin Boland, Rachel Fitzpatrick, Iracema Leroi, Joanna Coast

**Affiliations:** a School of Medicine, Trinity College Dublin, College Green, Dublin 2, Dublin, Ireland; b Global Brain Health Institute, Trinity College Dublin, College Green, Dublin 2, Dublin, Ireland; c Health Economics & Health Policy (HEHP@Bristol), Bristol Medical School, University of Bristol, Bristol, United Kingdom

**Keywords:** Dementia, Quality-of-life, Outcome measurement, ICECAP, Self-reporting, Lewy body dementia, Capability approach, Response process validity

## Abstract

**Background:**

Capability measures offer theoretical advantages for assessing wellbeing in health economics and outcomes research, yet their feasibility for self-reporting by people living with dementia remains to be established. This exploratory study compares the feasibility of self-reporting on capability versus health-focused quality of life measures among people living with mild-tomoderate dementia, using response process validation.

**Methods:**

Twenty-three community-dwelling participants living with Alzheimer’s disease (*n* = 11) or Lewy body dementia (*n* = 12) completed four measures using concurrent think-aloud cognitive interviewing: two capability measures (ICECAP-O, ICECAP-SCM) and two health-focused measures (QoL-AD, and AQoL-4D). Errors were coded using Tourangeau’s four-stage response model (comprehension, retrieval, judgement, response). Completion times, support requirements, and error patterns were compared across measures and diagnostic groups.

**Results:**

Capability measures showed higher error rates (13.7-18.3%) than health-related measures (5.1-8.4%) despite shorter completion times. Comprehension errors (51.2% of all errors) were most common across all measures, particularly for abstract concepts like *dignity* and *doing things that make you feel valued*. Retrieval errors were rare (4.9%), suggesting memory is not the primary barrier. Diagnostic groups showed distinct error profiles. Participants living with Lewy body dementia showed higher rates of comprehension errors (57.4% vs 42.9%), whilst participants living with Alzheimer’s disease showed more response-selection difficulties (25.7% vs 14.9%).

**Conclusions:**

While self-reporting remains feasible in mild-to-moderate Alzheimer’s disease and Lewy body dementia, capability measures pose greater cognitive demands than health-focused measures, primarily due to comprehension of abstract concepts and distinguishing capability from functioning rather than memory retrieval. Measures should be validated for specific dementia subtypes, and hybrid approaches anchoring capability judgements in concrete experiences warrant exploration. This exploratory study provides preliminary response process validity evidence to inform measure selection in dementia research and clinical practice, ensuring people living with dementia can meaningfully participate in health economics and policy decisions that affect their care.

## 1 Introduction

Dementia currently affects around 55 million people worldwide, and this number is expected to rise to over 139 million by 2050 (World Health Organisation, 2025). The impact of dementia extends far beyond the individuals diagnosed. The global economic cost was estimated at $1.3 trillion in 2019, and this figure is projected to double to $2.8 trillion by 2030 (Alzheimer’s Disease International, 2022). These rising numbers place immense strain not only on healthcare systems and national economies but also on social and long-term care settings, as well as on informal and family carers.

In health economics and outcomes research (HEOR), measuring outcomes is essential for evaluating the effectiveness of interventions, guiding resource allocation, and informing policy decisions. Economic evaluations of interventions for dementia have traditionally used generic preference-based measures such as EQ-5D (EuroQol 5-Dimension)(Herdman et al., 2011), SF-6D (Short Form 6-Dimension)(Brazier et al., 2002), AQoL-4D (Assessment of Quality of Life 4-Dimension) (Hawthorne et al., 1999)), or condition-specific health-related quality-of-life (HRQoL) measures such as QoL-AD (Quality of Life in Alzheimer’s Disease)(R. G. Logsdon, Gibbons, L.E., McCurry, S.M., Terri, L., 1999), DEMQOL (Dementia Quality of Life) (Smith et al., 2007)), and other (Li et al., 2018). Whilst condition-specific measures capture aspects of wellbeing relevant to particular populations, they remain grounded in health-focused frameworks that emphasise functioning and health states. Recent evidence suggests these measures may not fully reflect broader aspects of wellbeing that people living with dementia value, such as autonomy, security, and opportunities for choice and control, domains that extend beyond health status (Engel et al., 2020; Kinchin et al., 2025). As international policies like the World Health Organization Global Action Plan on Dementia (WHO, 2017) increasingly emphasise person-centred, holistic care, there is growing recognition that current HRQoL instruments may need to be complemented by alternative evaluative frameworks that better reflect the lived experiences and priorities of people living with dementia (Kinchin, 2025). Person-centred care prioritises the individual’s preferences, values, and autonomy in care decisions, while holistic care addresses physical, psychological, and social dimensions of wellbeing rather than focusing solely on disease management (Brooker, 2003; Kitwood, 1997; WHO, 2017)

### 1.1 The capability approach

The capability approach, developed by Sen (1993) and increasingly used in HEOR, offers an alternative or complementary approach. Unlike HRQoL instruments, which focus on functioning (what people do), the capability approach examines what people are able to do or be, their freedoms and opportunities (Sen, 1993). Traditional functioning-based measures like the EQ-5D, for example, assess domains such as mobility, self-care, usual activities, pain/discomfort, and anxiety/depression, focusing on what individuals actually do or experience. In contrast, capability measures assess whether people have the opportunity and freedom to achieve states of being they value. By shifting the focus from observed outcomes to potential capabilities, this approach makes it possible to capture aspects of wellbeing that functioning measures may miss. For example, a person living with dementia may be physically able to leave their home but lack the confidence or support to do so or may value having the choice to engage in social activities even if they choose not to. This distinction is particularly important for populations like older adults or those living with dementia, where wellbeing depends not only on what they can physically do but also on psycho-social factors such as their sense of autonomy, security, and opportunity.

A suite of capability measures for use in economic evaluation has been developed by Professor Joanna Coast and colleagues for different populations and contexts. For example, the capability measure ICECAPO (designed for older adults) assesses wellbeing across five domains, including attachment (love and friendship), security (thinking about the future without concern), role (doing things that make you feel valued), enjoyment (enjoyment and pleasure), and control (independence) (Grewal et al., 2006). Similarly, the ICECAP-Supportive Care Measure (ICECAP-SCM) extends to seven domains relevant to end-of-life contexts, including love and affection, physical suffering, emotional suffering, dignity, being supported, being able to have a say, and being able to prepare (Sutton and Coast, 2014).

### 1.2 The gap: can people with dementia self-report on capability measures?

Studies have shown that ICECAP-O measures are valid and reliable for use with community-dwelling older adults, including those with mild cognitive impairment or early dementia (Bibi et al., 2022; Makai et al., 2014; Nyman et al., 2021). Yet, the feasibility of self-completion by people living with dementia remains underexplored, with most research in dementia continuing to rely on proxy responses. Where self-report has been attempted, agreement between proxies and the person is often only moderate, especially for less visible aspects of wellbeing like enjoyment or control (O’Shea et al., 2020).

Even among older adults without cognitive impairment, studies using cognitive interviews have highlighted the difficulty of distinguishing between what someone is “capable” of doing and what they “actually” do (Al-Janabi et al., 2013). In capability-based assessments, questions that probe an individual’s abilities often employ abstract language and complex ideas, which can be challenging to interpret.

This challenge may be amplified for people living with dementia, whose cognitive abilities decline in different domains and at different rates. For example, in Alzheimer’s disease, memory loss is usually the first and most prominent symptom, while attention and language are relatively preserved in the early stages (e.g. Zvěřová (2019)). In contrast, dementia with Lewy bodies may begin with problems in attention, visuospatial skills, and executive function, along with marked fluctuations in cognition (e.g. McKeith et al. (2017)). Research suggests that these fluctuations in Lewy body dementia can lead to greater variability in how people respond to questionnaires, raising the possibility that capability-based questions may be difficult for some groups.

### 1.3 The present study

While our recent commentary (Kinchin et al., 2025) demonstrated conceptually that capability measures may better capture what people with dementia value, this argument requires empirical evidence. Specifically, we need to know: (1) whether people with dementia can feasibly self-report on capability measures; (2) whether capability measures impose different cognitive demands than health-focused measures; and (3) whether feasibility differs across dementia types with distinct cognitive profiles. This exploratory study provides that evidence through systematic response process validation.

Building on the work of Al-Janabi et al. (2013), who used Tourangeau’s four-stage model of self-reporting, including comprehension, retrieval, judgement, and response selection (Tourangeau et al., 2000), this study addresses three interconnected research questions.

1. Do capability measures differ from health-focused measures in self-report feasibility? We compare completion rates, times, and support requirements between capability measures (ICECAP-O, ICECAP-SCM) and health-focused measures: one dementia-specific (QoL-AD) (R. G. Logsdon et al., 2002; R. G. Logsdon, Gibbons, L.E., McCurry, S. M., Terri, L., 1999) and one generic preference-based instrument (AQoL-4D) (Hawthorne et al., 1999).
2. What types of cognitive errors occur during self-completion, and do these differ between measure types? Using concurrent think-aloud methods, we systematically code comprehension, retrieval, judgement, and response errors to identify which measures impose greater cognitive demands.
3. Do feasibility and error patterns differ between Alzheimer’s disease and Lewy body dementia? Given the distinct cognitive profiles of these conditions, we examine whether different measures are better suited to different dementia types.

Together, these analyses explore which types of quality of life measures may be most feasible for self-reporting by people living with dementia, addressing the premature shift to proxy reporting and ensuring that the perspectives of people living with dementia remain at the heart of decisions about care, interventions, and policies designed to enhance their wellbeing.

## 2 Methods

This study was reported in accordance with the COREQ 32-item checklist (Tong et al., 2007). A completed checklist mapping items to manuscript sections is available in Supplementary materials.

### 2.1 Study setting and recruitment

Participants were purposively recruited to represent two major diagnostic groups, Alzheimer’s dementia and Lewy Body dementia. The recruitment strategy considered demographic characteristics such as age, sex and geographical location (urban and rural). To reach participants outside Dublin, two complementary recruitment pathways were used:

1. Memory and movement disorder clinics at two major hospitals in Dublin (urban). Clinicians introduced the study to eligible patients with clinically confirmed diagnoses and provided information leaf-lets. Interested patients contacted the research team directly or consented to be contacted (name, email, phone).
2. Local dementia support groups facilitated by third-sector organizations with national reach (urban and rural, including regional/rural areas). Support group facilitators reached out to potential participants through their networks.

We monitored diagnostic group and symptom severity during sampling to mitigate imbalance. Upon initial contact, the research team provided detailed study information to potential participants, explained the consent form, obtained informed consent, and arranged interviews at a time and location convenient for the participant.

Eligible participants were those with a clinical diagnosis of Alzheimer’s disease or Lewy Body dementia, who had capacity to consent, were fluent in English (given the English language questionnaire under consideration), and were willing to participate. A detailed study protocol has been published elsewhere (Kinchin et al., 2022).

### 2.2 Data collection

A concurrent think-aloud cognitive interviewing approach informed by the Tourangeau four-stage response model was used to structure probes (Tourangeau et al., 2000). Immediately following each interview, field notes containing interviewer observations were taken. A semi-structured interview guide (pilot-tested with the Lived Experience Group at the Global Brain Health Institute) covered diagnostic journey, perceptions of a good life and self-completion of four outcome measures (detailed below) and did not change during data collection.

Interviews were conducted between September 2022 and February 2023, either in person or via video call based on participant preference. In-person interviews took place in participants’ homes, clinic rooms or University campus rooms ensuring privacy. Only the participant, interviewer, and where applicable care partners were present. Where care partners were present for support, they were instructed not to provide answers on behalf of participants unless asked. Each interview lasted approximately 60-90 min, was audio-recorded, and transcribed verbatim. Transcripts were not returned to participants for comment or correction. No repeat interviews were conducted. Participants received no incentive.

All interviews were conducted by a female researcher (IK) with training in qualitative interviewing and think-aloud methods, and prior experience working with people living with dementia. No prior relationship existed between the interviewer and participants. At first contact, participants were informed of the interviewer’s role and the study aims. The research team reflected on its assumptions about HRQoL and capability measures and mitigated potential bias through use of a semi-structured interview guide, standardised prompts, and multi-coder consensus. Data collection was judged to be sufficient when themes were felt to be adequately captured and when no new error types or probe-elicited issues emerged across three consecutive interviews in each diagnostic group.

Each interview began with a briefing on the study objectives, format, and consent procedure. For in-person interviews, written consent was obtained, while video call interviews required audio-recorded verbal consent. The initial discussion focused on participants’ experiences, from first noticing symptoms through to receiving their dementia diagnosis, followed by their reflections on what constitutes a good life with results published elsewhere (Conlon et al., 2025; Kinchin et al., 2025). Participants then completed adapted versions of ICECAP-SCM and ICECAP-O (provided in Supplementary materials, Tables 1 and 2), and the original versions of Quality of Life in Alzheimer’s Disease (QoL-AD)/AD-5D, and Assessment of Quality of Life (AQoL-4D) (Table 1).

The administration sequence of the four outcome measures was randomised across participants using Microsoft Excel to minimise order bias and control for fatigue effects on response quality. The distribution of measures completed last was ICECAP-SCM (*n* = 4), ICECAP-O (*n* = 6), QoL-AD (*n* = 4), and AQoL-4D (*n* = 9), although one participant was unable to complete the AQoL-4D due to fatigue.

To aid interpretation of the findings, dementia severity was also classified using the Dementia Communication Difficulties Scale (DCDS; Murphy et al., 2007) and the General Practitioner Assessment of Cognition (GPCOG; Brodaty et al. (2002). DCDS scores defined stages as mild/early (0–10.5), moderate (11–19.5), and advanced/late (20–39). On the GPCOG, patient scores above 8 indicated intact cognition and scores below 5 signalled impairments, while for the informant version, scores ≤3/6 suggested cognitive impairment. The DCDS and GPCOG were administered by the interviewer after the interview session. Prior to data collection, the interviewer received training on instrument purpose, standardised administration, scoring, and interpretation. A brief scoring guide was used in the field to ensure consistency. Borderline cases were double-scored and discrepancies resolved by consensus.

Using concurrent think-aloud methods, participants were encouraged to verbalise their thought processes in real-time whilst completing outcome measures. This approach was chosen over retrospective think-aloud to minimise reliance on memory, a key consideration given the cognitive impairments associated with dementia. Prior to completing the outcome measures, participants were given a brief practice task to familiarise them with the think-aloud process. The interviewer provided gentle, non-directive prompts (e.g., what are you thinking right now? or can you tell me more about that?) during pauses and used open-ended, non-leading questions to elicit detailed responses and clarifications without influencing their answers. We recognised that concurrent think-aloud can be cognitively demanding, particularly for people living with dementia (Jaspers et al., 2004). To mitigate this, the interviewer monitored for signs of fatigue or distress, and interviews were concluded early if participants appeared unable to engage. The randomisation of measure administration order also helped distribute any cumulative cognitive burden across different measures. If participants were unable to engage or deemed tired, the interview was concluded early. Completion time was recorded for each measure. Transcripts and field notes were managed and coded in Excel.

### 2.3 Data analysis

Initially, four independent raters conducted preliminary data extraction using a staggered approach, in which three transcripts were analysed (EB, RF, DR, IK) to establish a consistent understanding and methodology, with guidance from an experienced reviewer (JC). Early coding treated scored struggles/uncertainty and carer influence as errors. On review, we determined these represent distinct response features and reclassified them into separate categories. A new scoring framework was developed and applied de novo to all interviews by an independent rater (JC), with the final dataset confirmed in a consensus meeting (IK). While intercoder agreement was not calculated, all coding decisions were reached through iterative discussion and consensus amongst raters, with disagreements resolved through team meetings and, where necessary, majority decision. This consensus-based approach prioritised in-depth consideration of each error classification over statistical measures of reliability. Illustrative quotations are presented in the Results, attributed by participant ID presented separately for each diagnostic group.

Errors were categorised into four types based on the Tourangeau model (Tourangeau et al., 2000).

1. *Comprehension*: Difficulties understanding the intended meaning of questions, including any misunderstanding of a word or phrase.
2. *Retrieval*: Challenges in recalling relevant information from memory, such as miscalculating the time frame stated in the question.
3. *Judgement*: Problems in appropriately applying retrieved information to formulate answers, such as misunderstanding what is being asked or misinterpreting the question.
4. *Response*: Answers that were inconsistent with personal experiences, inconsistent with previous responses, fell outside the expected options, or appeared to be socially desirable.

The following rules were adapted from the work of Froggatt et al. (2020) and used to guide the classification of errors.

- *No error identified*: If none of the raters identify an error, no error will be recorded.
- *Unanimous agreement*: If all raters agree on a particular error type, it will be recorded as an error.
- *Partial agreement*: If one or two raters identify an error, the raters will engage in a discussion to reach a consensus on the final classification.
- *Disagreement on error type*: If all raters identify an error but disagree on its nature, they will discuss the issue to come to a final decision.
- *Majority decision*: If the raters are unable to reach a consensus, the error classification will be determined by the majority view.

In addition to the four error types outlined by the Tourangeau model (Tourangeau et al., 2000), two further categories were recorded to capture relevant participant experiences that were *not* classified as errors.

- *Struggle*: This category, developed in earlier *ICECAP* studies (Al-Janabi et al., 2013), refers to general difficulties with question completion, such as uncertainty or hesitancy when considering different answers. Importantly, *struggle* does not imply that an error was made but highlights when participants experienced difficulty despite ultimately providing an appropriate response.
- *Uncertainty*: This was an additional category developed for this work, which referred to instances where participants expressed a loss of confidence (not linked to comprehension issues) or sought reassurance. While uncertainty may overlap with struggle, it was treated as a separate category to capture moments where participants were unsure of their responses, even if they were ultimately correct.

Additionally, two other factors that appeared to influence participant responses were recorded.

- *Carer influence*: Instances where carers influenced participants’ responses, resulting in agreement, disagreement, or a change of answer. While this was not categorized as an error, it was recognised as an important factor in response patterns.
- *Articulation/voice impairment*: Cases where participants struggled to articulate their responses clearly due to mumbling or voice impairments. While not classified as an error, this was recorded as it impacted response clarity.

By distinguishing between errors, participant difficulties, and other influencing factors, this bespoke framework aimed to provide a comprehensive understanding of the challenges people living with dementia may face when self-reporting their quality of life.

Given the qualitative focus of this study and the sample size, our primary analyses compared error patterns between the two diagnostic groups (Alzheimer’s disease vs. Lewy body dementia) and across the four measures. We did not conduct stratified analyses by dementia severity, age, symptom duration, or care partner presence, as the sample size would not support reliable subgroup comparisons.

### 2.4 Error rate calculation

Error rates were calculated as the proportion of errors relative to the total number of opportunities for error. Importantly, this method assumes that each participant can make only one prominent error per item. This ensures that the error rate reflects the distinct number of errors rather than multiple errors from the same participant within a single item.

The error rate for each measure was calculated using the following formula:

Errordensityrate=(Totalerrors/(Numberofitems×Numberofparticipants))×100

For example, the ICECAP-O measure includes 5 items and was administered to 23 participants, resulting in 115 total opportunities for error (5 items × 23 participants). Within these 115 opportunities, 21 observed errors were recorded. The error rate was calculated as follows: (21/115) × 100 = 18.3%.

This calculation method was applied consistently across all four measures.

- ICECAP-SCM (7 items, 161 opportunities for error)
- ICECAP-O (5 items, 115 opportunities for error)
- QoL-AD (13 items, 299 opportunities for error)
- AQoL-4D (12 items, 276 opportunities for error)

Error rates were calculated for each measure both overall and separately for participants with Alzheimer’s disease and Lewy Body dementia to facilitate comparison of performance patterns across these two major diagnostic groups.

### 2.5 Interviewer observations

Following each interview, the interviewer (IK) recorded reflexive field notes documenting observations of participant responses, behaviours, and difficulties encountered during assessment completion. These notes captured patterns relating to confidence and validation-seeking behaviours, challenges with comprehension (particularly abstract concepts), memory-related issues, emotional reactions, discrepancies between self-reports and observed circumstances, and care partner influence on the response process.

Field notes were reviewed iteratively throughout data collection, allowing emerging patterns to inform subsequent observations. Following completion of data collection, field notes were reviewed alongside the coded error and think-aloud data at team consensus meetings. This integrated approach enabled triangulation of interviewer observations with participant verbalisations and documented errors, strengthening the credibility and contextual understanding of findings. Recurring patterns and illustrative examples were identified through team discussion, with observations grounded in documented field note evidence.

### 2.6 Ethics

This study was approved by the Joint Research Ethics Committee of St James’s Hospital and Tallaght University Hospital on April 11, 2022. All participants provided their own informed consent for audio-recording and for the use of anonymised quotations.

## 3 Results

### 3.1 Participant characteristics

Twenty-three participants formed the analytic sample, including 12 people living with Lewy Body dementia and 11 with Alzheimer’s disease. Participant demographics are outlined in Table 2.

### 3.2 Completion patterns and support required

On average, participants completed the capability measures (ICECAP-SCM and ICECAP-O) faster than the QoL-AD and AQoL-4D (Table 3). The ICECAP-SCM and ICECAP-O took an average of 7.9 (SD 4.4) and 6.9 (SD 2.9) minutes respectively, while the QoL-AD and AQoL-4D required 9.2 (SD 5.0) and 11.3 (SD 5.0) minutes respectively.

Descriptively, completion times appeared to vary by dementia type, with participants with Alzheimer’s disease tending to complete all measures more quickly than those with Lewy Body dementia. This difference was most pronounced for the ICECAP-SCM, where participants with Alzheimer’s averaged 6.6 (SD 3.6) minutes compared to 9.0 (SD 5.0) minutes for those with Lewy Body dementia. However, given the qualitative focus of this study and the lack of statistically significant differences when exploratory tests were conducted (*p* > 0.05 for all comparisons), we present these as descriptive patterns rather than inferential findings. We acknowledge that these patterns may be influenced by unmeasured confounders including dementia severity, age, and symptom duration, which we were unable to control for given the sample size.

Support from care partners and/or interviewers was required across all measures, though the extent varied. While self-reporting appeared feasible for most participants, ICECAP-O required the least support (4 instances) and AQoL-4D required the most support (14 instances) (Table 3).

### 3.3 Error patterns and diagnostic group differences

The analysis identified 82 errors across all measures: 47 (57.3%) from participants with Lewy Body dementia and 35 (42.7%) from those with Alzheimer’s disease (Table 4). Overall error rates ranged between 5.1 and 18.3% across all measures (Table 3). Traditional health-related measures had lower error rates (QoL-AD: 8.4%; AQoL-4D: 5.1%) than capability measures (ICECAP-SCM: 13.7%; ICECAP-O: 18.3%).

Comprehension was the most common error type, accounting for 42 instances across both diagnostic groups, though more frequent in the Lewy Body dementia group (27 instances) than the Alzheimer’s group (15 instances). ICECAP-O had the highest comprehension error rate (10.4%), with participants frequently asking for clarification, for example: *‘Um, I don’t understand that question, I’m sorry now*’ (P8).

Judgement error affected both groups equally (10 errors each), representing challenges in making evaluative decisions. This was most evident in ICECAP-O (5.2% error rate), where participants’ verbal explanations often contradicted their categorical responses. For example, on ‘*love and friendship*’, P14 vacillated between adjacent categories: “*Okay. I will say almost all … oh yeah, I’d say … a lot …*,” then, when asked “*A lot or all that you want?*,” replied “*All that I want*,” while noting non-criterion considerations: “*It’s very arrogant … Don’t tempt fate*.” On ‘*independence’*, partner input appeared to shape the final choice, after moving through several options (“*In many things … in a few things*”), the care partner interjected “*In a few things*,” which P14 then endorsed, “*I think it’s representative*.”

Participants with Lewy Body dementia seemed to ‘struggle’ with the assessment process considerably more (20 vs 3 instances), for example: *‘I don’t know what I’m answering’* (P23). Participants with Alzheimer’s showed a slightly higher frequency of response-selection difficulties (9 vs 7 instances), by addressing tangential aspects rather than the core question. However, this difference was modest.

Error patterns also varied between measures. Traditional measures demonstrated more balanced error distribution across items (Supplementary materials, Tables 3–5), with no single item accounting for more than 20% of total errors. In contrast, capability measures showed concentration of errors in specific items, with ICECAP_O *‘doing things that make you feel valued’* accounting for 61.9% of total errors and ‘*dignity*’ in ICECAP-SCM accounting for 40.9% of total errors (Table 3).

### 3.4 Cognitive processing patterns

Cognitive processing patterns showed that retrieval errors were rare (only 4 instances in total), suggesting participants could access relevant information once they understood questions. However, making evaluative judgements remained equally challenging for both groups (10 errors each). These judgement errors typically manifested as discrepancies between participants’ verbal explanations and their chosen categorical responses.

### 3.5 Measure-specific performance

#### 3.5.1 ICECAP-O

The ICECAP-O showed the highest error rate (18.3%), regardless of the diagnostic group (Lewy body dementia: 18.3%; Alzheimer’s: 18.2%), with one question proving particularly problematic, i.e., *‘doing things that make you feel valued*’. Participants exhibited comprehension and response-selection difficulties, for example conceptual misalignment (P11), explicit parsing/wording challenges (P12), evolving or externally anchored interpretations that sought confirmation (P6), and uncertain access to the construct (P7).

P11: *I don’t really need to be valued do you know, I don’t, I don’t particularly, I don’t particularly want to be valued. My notion of value in my head would be the one that keeps me going. As I’m sure we all do, you know, we all do, sit down, say to yourself after whatever happened you say, why did you do that or why did you say that? That was ridiculous, you know what I mean?*

P12: *The wording of the question … I’d have to think about it. I’d have to think about it. I’d find the wording of that just a little bit, just a little bit confusing. It’s something I’d have to spend time thinking about*.

P6: *The answer is back from the people I’m talking to isn’t it? If they think they value me. I can’t OK well, all that make me feel valued, things that make you feel valued. See I do all the housework. I do everything I can. Is that then an answer?*

P7: *When do I, I don’t know that I feel valued um, do I? No I’d say many, not all. Yeah*.

Even with interviewer (I) scaffolding (P9), several responses drifted from the intended action-oriented capability toward broader feelings of being valued.

P9: *What, um let me see … What makes me feel … Well like I have a lot of wonderful friends and people in my life so that’s you know a huge value to me*.

I: *And if you want to, are you able to do things that make you feel valued, for example all of the things or many or a few?*

P9: *I think so yeah, yeah … I’m just trying to really understand the question you know. Doing things that make you feel valued … I think that’s it’s, it’s a difficult question really to me. I feel, like I do feel valued you know, all of those things so um yeah I can’t*.

#### 3.5.2 ICECAP-SCM

The ICECAP-SCM had the second-highest error rate (13.7%). Comprehension was particularly challenging for the Lewy Body dementia group, with an error rate of 14.3% compared to 5.2% in the Alzheimer’s group. The concept of ‘*dignity*’ emerged as one of the most challenging on the ICECAP-SCM for both groups, generating 9 distinct errors across three categories: comprehension, judgment, and retrieval. Some participants found it difficult to grasp the meaning, asking: “*Well, what does dignity stand for?*” (P6). Another participant’s response revealed their struggle to define the concept: “*Dignity, uh I suppose in one way it would be, um, dignity, yeah it’s probably just being able to manage yourself, um, yeah. I suppose that, that’s … I think dignity is more about sort of … It’s more about your character really, I think, you know. I don’t know. I don’t really, yeah …”* (P9). The challenge was perhaps most clearly expressed by a participant who simply stated: *“I, I don’t know what that means really”* (P23). Similar to feedback received about the ICECAP-O *‘doing things that make you feel valued’*, participants suggested that providing a clear definition of ‘*dignity*’ would help them better understand and answer this question.

#### 3.5.3 QoL-AD

The QoL-AD maintained a relatively moderate error rate (8.4%), contrasting with capability measures through its emphasis on current functioning rather than potential capabilities, which appeared to make it more accessible for participants to understand and answer. Though the measure performed well overall, the difference between diagnostic groups appeared, participants with Lewy Body dementia showing a slightly higher error rate (9.6%) compared to those with Alzheimer’s (7.0%). In the QoL-AD, questions addressing memory, ability to do things for fun, and self-evaluation proved difficult, particularly for those with Lewy Body dementia. The question about *‘ability to do things for fun’* generated five errors, mainly involving comprehension errors. An interesting pattern emerged where participants would describe their hobbies and enjoyable activities yet rate their ability to engage in these activities as poor, suggesting they may have misinterpreted the question. The need for clarification was evident in participants’ responses, such as one who simply echoed, *“Do things for fun”* (P23), and another who admitted, *“I don’t know. I can’t think of what it is”* (P16). A particularly revealing exchange occurred when one participant stated, *“I don’t do a lot of things*,*”* but then rated their ability as ‘Excellent’ when presented with the rating scale (P17).

The question asking participants to evaluate themselves *‘as a whole’* also proved challenging, resulting in four errors related to judgment and comprehension. Participants’ confusion was evident in their responses, such as “*You mean how do I feel in myself or about* …” (P1) and “*I don’t understand that question*” (P23). One participant offered insightful criticism, suggesting that the question was too constraining: “*Assessing quality of life, it’s too rigid. It’s too specific in terms of x, y, and z. If you allowed more space for a person just to ramble, philosophize, talk about religion, talk about things that really capture them, incidents, not money, you see*” (P10).

#### 3.5.4 AQoL-4D

The AQoL-4D, despite being the longest measure in this study (12 items), had the lowest overall error rate (5.1%), again likely due to its focus on concrete functioning. Unlike previous measures, participants with Alzheimer’s showed a higher error rate (6.8%) compared to participants with Lewy Body dementia (3.5%). While participants handled straightforward physical health questions well, they seemed to encounter some difficulties with items about relationships and daily activities. Error rates for these items were 1.5% and 0.7% for participants with Alzheimer’s and Lewy Body dementia respectively.

The section on family relationships also proved somewhat problematic, generating three errors across multiple categories: comprehension, retrieval, and judgment. For example, one participant said, “*That’s a hard one because I don’t know what way to do this*” (P4). Another participant offered feedback: “*Thinking about your health and your relationship with your family, your participation in the family is unaffected by my health, there are some parts of my family role I cannot carry out. That one is hard to answer accurately. It’s subtle*” (P10). Participants suggested several improvements, including specifying time frames, focusing on immediate family relationships, and breaking down complex questions into simpler components. Many asked for overall simplification to enhance clarity and ease of response.

Overall participants needed the interviewer’s help to understand and navigate the response options. Though they appreciated the questionnaire’s directness and range of response choices, many initially found the format confusing and wording lengthy. Despite these difficulties, participants emphasised the importance of including self-care questions in the assessment.

### 3.6 Interviewer observations

#### 3.6.1 Participant confidence

Participants commonly sought validation of their responses, pausing to ask “*Is that right?*” or looking to the interviewer or care partner for confirmation. Several used humour to deflect from challenging questions, such as one participant (P14) who laughed when asked about control over their life, saying “*Well, I never had much control even before this!*” Female participants generally showed greater willingness to discuss emotional topics openly, whilst some older male participants gave brief or deflecting responses to questions about feelings or relationships.

#### 3.6.2 Question comprehension and memory

Participants struggled with abstract concepts (dignity, independence, attachment), frequently requesting clarification: “*What do you mean by that?*” or “*Can you give me an example?*” Concrete questions with clear response options were answered with greater ease. Memory limitations manifested in several ways, participants sometimes lost track of questions whilst developing answers, showed stronger recall for historical events than recent experiences, and frequently selected the most recently presented response option (recency effect). Fatigue became evident as interviews progressed, with increased hesitation and longer response times affecting performance on later instruments.

#### 3.6.3 Emotional and sensitive topics

Questions about future planning and end-of-life preparations triggered visible distress (tearfulness, voice changes) among some participants. However, many valued being asked whilst retaining cognitive capacity, with one participant (P1) stating: “*I’m glad you’re asking me this now, while I can still tell you what I want*.” Questions about diminished independence or self-worth also caused distress, with participants becoming quiet or changing tone. Financial matters were particularly problematic for those who had relinquished monetary control, with one participant (P19) responding: “*I don’t really know anymore [care partner’s name] handles all that now*.” Participants found relationship questions challenging, requiring clarification about whether these referred to family, friends, care partners, or all connections collectively.

#### 3.6.4 Self-perception

Many participants displayed discrepancies between subjective experiences and objective circumstances. Some rated themselves positively despite observable functional limitations (e.g., P5 rated independence highly despite requiring substantial assistance), whilst others showed acute awareness of declining capabilities. Questions about self-care revealed variable awareness of support needs, with some participants rating themselves as independent in areas where care partners later indicated significant assistance was provided. Daily activity questions led to inconsistent responses, with participants interpreting scope differently (e.g., household tasks ranging from basic cleaning to complex home maintenance).

#### 3.6.5 Care partner presence and influence

Care partner presence potentially influenced participant responses in various ways. Some care partners interjected to correct or supplement responses before participants finished speaking. In one interview (P12), the participant visibly sought confirmation from their care partner before answering, then offered a tentative reply: “*I think … maybe* …” whilst continuing to look at them. Whilst some care partners provided facilitative support to help articulate thoughts, others appeared to influence responses more directly by prompting specific answers or expressing disagreement. Even silent presence may have affected participants’ willingness to express certain views, particularly regarding relationship quality or care satisfaction.

## 4 Discussion

This study provides the first response process validityevidence comparing the feasibility of self-reporting on capability versus healthfocused quality of life measures in mild-to-moderate Alzheimer’s disease and Lewy body dementia. The findings reveal meaningful differences in measure performance, diagnostic-specific error profiles, and practical implementation considerations with implications for measure selection in dementia research and practice.

While capability measures (adapted versions of ICECAP-SCM and ICECAP-O) were completed faster than traditional health-related measures (QoL-AD, AQoL-4D), they showed higher error rates (13.7-18.3% vs 5.1-8.4%). This inverse relationship between completion time and accuracy challenges the assumption that brevity reduces cognitive burden. Rather than indicating cognitive ease, faster completion likely reflects difficulty engaging with abstract capability constructs, participants may have provided responses without full comprehension, whereas longer completion times for HRQoL measures suggest more careful consideration of concrete questions about current functioning. Participants with Lewy body dementia required more time overall, particularly for ICECAP-SCM (9.0 vs 6.6 min for Alzheimer’s disease), reflecting the additional cognitive load imposed by executive and attentional deficits when processing abstract, future-oriented content.

The low frequency of retrieval errors (4.9% overall) suggests that memory difficulties may not be the primary barrier to completing quality of life assessments. Instead, comprehension (51.2% of errors) and judgement (24.4%) errors were more common, indicating that clear language and response formats may matter more than memory demands. The superior performance of traditional HRQoL measures likely reflects their grounding in current functioning, the QoL-AD’s focus on present functioning and the AQoL-4D’s physical health items proved more accessible than capability-oriented questions, aligning with previous research emphasizing the importance of plain language in dementia research (Collins et al., 2022; Conway et al., 2023).

Our error rates for ICECAP-O (18.3%) and ICECAP-SCM (13.7%) were substantially higher than those reported in cognitively healthy populations (Horwood et al. (2014): 5%; Bailey et al. (2016): 3.9%; Nwankwo et al. (2022): 5.7%), with comprehension errors predominating across all studies. For ICECAP-O, the ‘role’ attribute was most challenging, while for ICECAP-SCM, the ‘dignity’ item proved difficult. These patterns suggest that abstract capability items are challenging even for healthy adults, yet pose substantially greater difficulties for people living with dementia, likely reflecting both the inherent complexity of capability concepts and the additional cognitive demands associated with dementia. (Grewal et al., 2006).

### 4.1 Capability versus functioning

A recurring source of difficulty was the distinction between capability (what a person is able to do or be, given their opportunities) and functioning (what they currently do or are). This exploratory work suggests that, for people living with dementia, mapping from present experience to a broader notion of capability is cognitively demanding and can blur with functioning, contributing to comprehension errors. This finding aligns with Horwood et al.’s observation that participants struggled to distinguish capability from functioning, and it helps explain the higher error rates on capability measures despite shorter completion times. Practically, this suggests a need to clarify the capability construct in item wording (e.g., brief definitions, concrete examples) or to consider hybrid formats that anchor capability judgments in recent, concrete experiences.

### 4.2 Diagnostic group differences

The findings further reveal distinct error profiles between people living with Alzheimer’s disease and Lewy body dementia. Comprehension errors were higher in the Lewy body dementia group (57.4% vs. 42.9%), aligning with evidence that prominent executive and attentional dysfunction can impair the evaluation and application of information during judgment (McKeith et al., 2017; Taylor et al., 2020; Walker et al., 2015). By contrast, the Alzheimer’s group showed more response errors (25.7% vs. 14.9%), consistent with semantic and language difficulties that can hinder translating judgments into categorical responses at the response stage (McKeith et al., 2017; Taylor et al., 2020; Walker et al., 2015).

Participants living with Lewy body dementia also appeared to ‘struggle’ with the assessment process considerably more than those with Alzheimer’s disease (20 vs 3 instances), with comments such as *‘I don’t know what I’m answering*’ (P23) reflecting broader difficulties engaging with abstract constructs. This pattern was particularly evident for ICECAP-SCM, where participants living with the Lewy body dementia showed higher error rates (19.0% vs. 7.8%). This difference likely reflects the measure’s dual cognitive demands as it requires both future-oriented thinking and engages decision-making and planning capacities that are disproportionately affected by the executive dysfunction characteristic of Lewy body dementia.

Interestingly, diagnostic group performance varied by measure. Participants living with Lewy body dementia showed higher error rates on QoL-AD (9.6% vs. 7.0%), while participants with Alzheimer’s disease struggled more with AQoL-4D (6.8% vs. 3.5%). This pattern may reflect the fact that QoL-AD was developed specifically for Alzheimer’s, potentially making its language and constructs more accessible to this group, while the AQoL-4D’s emphasis on complex daily activities and social roles may be more challenging for those with the memory and language difficulties characteristic of Alzheimer’s disease.

However, these diagnostic group differences should be interpreted cautiously in light of sample heterogeneity. While we stratified by diagnosis, we combined mild and moderate dementia stages throughout our analyses due to sample size constraints. This means that observed differences between diagnostic groups may partly reflect differences in severity distributions (LBD: FAST 2-6; AD: FAST 2-4) or other participant characteristics including age (range: 55-85 years), symptom duration (1-19 years), and care partner presence during interviews (61% of interviews). The higher error rates in the Lewy body dementia group, for example, could reflect either diagnosis-specific cognitive profiles, the slightly broader severity range in this group, or the interaction between diagnosis and disease progression. Similarly, the longer completion times observed in the Lewy body dementia group, particularly for ICECAP-SCM (9.0 vs 6.6 min), may reflect not only the cognitive demands of abstract, future-oriented content but also individual variation in processing speed and fatigue.

Despite these interpretive limitations, the distinct error profiles across diagnostic groups suggest that cognitive phenotype matters for measure feasibility. Even seemingly simple quality of life measures can impose different cognitive demands depending on the underlying pattern of cognitive impairment. This has practical implications, i.e. measures should be validated and, where needed, adapted for specific dementia subtypes and disease stages, rather than assuming that validation in one dementia population generalizes to others. Future research with larger samples should examine whether specific participant characteristics (severity, age, symptom duration, care partner presence) moderate the relationship between diagnosis and measure feasibility, and whether certain measure types are systematically better suited to particular cognitive profiles.

### 4.3 ICECAP-SCM appropriateness and target population considerations

The ICECAP-SCM is designed for end-of-life contexts, which may help explain our results. Bailey et al. (2016) reported that people in earlier disease stages felt they had to “guess” future experiences when completing ICECAP-SCM items and preferred the ICECAP-A, pointing to a temporal misalignment between current lived experience and hypothetical end-of-life scenarios. This misalignment likely contributed to difficulties among our participants living with mild-to-moderate dementia, particularly given challenges with future-oriented thinking.

The higher error rate for participants with Lewy body dementia on ICECAP-SCM (19% vs. 7.8% for AD) likely reflects the measure’s dual cognitive demands, not only does it require future-oriented thinking, but many items (e.g., ‘making important preparations’) tap decision-making and planning capacities that are disproportionately affected by the executive dysfunction characteristic of LBD. The higher support requirements for ICECAP-SCM (12 instances) compared to ICECAP-O (4 instances) may further reflect not only this temporal misalignment with current experience but also the emotionally complex nature of end-of-life planning, which often necessitated care partner input and clarification.

Although discussions about future planning and end-of-life preparations triggered strong emotional reactions among some participants, many expressed that these conversations were crucially important to have during the early stages of their dementia journey, when they could still actively participate in decisions affecting their future care and quality of life. This highlights the complex tension between the emotional difficulty of such conversations and their profound importance for person-centred care.

### 4.4 Implications for practice

These findings have several practical implications. Traditional health-related measures performed better and may be more suitable for routine use, given their narrower focus and relative ease of completion, whereas capability measures likely require further adaptation for self-reporting by people living with dementia. The concentration of errors around more abstract concepts, *‘dignity’* (40.9%) and *‘valued activities’* (61.9%), suggests a need for clearer definitions, concrete examples, and possibly alternative response formats.

As a potential way forward, measures of wellbeing that emphasise functioning (what people do and experience) rather than capabilities (what people are free to do) may balance breadth with feasibility. That said, constructs such as dignity and role remain conceptually complex, even when framed as functioning, and measurement may still pose challenges. Interviewer observations reinforce the value of plain, concrete language, illustrative examples, and attention to cognitive fatigue. While some instruments have been developed for Alzheimer’s disease, measures should be validated and, where needed, adapted for each clinical context, including dementia subtype and disease stage; our findings suggest that Lewy body dementia may warrant distinct approaches.

### 4.5 Limitations and future directions

#### 4.5.1 Sample size and participant heterogeneity

This study’s sample of 23 participants, while appropriate for qualitative cognitive interviewing and response process validation, limits our ability to conduct stratified quantitative analyses by multiple participant characteristics. This heterogeneity limits our ability to disentangle diagnosis-specific patterns from severity-related effects, and observed differences should be interpreted cautiously. Similarly, we did not stratify by other potentially relevant characteristics including age group, symptom duration, or care partner presence during interviews.

The decision to combine mild and moderate dementia stages throughout the analyses was driven by the research focus on feasibility of self-report in mild-to-moderate dementia (as opposed to advanced dementia where self-report is typically not attempted) and primary interest in comparing diagnostic groups with distinct cognitive profiles. However, this means that observed differences between diagnostic groups may partly reflect differences in severity distributions or other unmeasured participant characteristics rather than diagnosis alone. Future studies should prespecify severity-stratified analyses and consider longitudinal designs to track how error profiles evolve with disease progression. Where feasible, incorporating additional cognitive markers (e.g., MoCA/MMSE, attention/executive composites) would enable sensitivity analyses to test the robustness of diagnosis effects after accounting for severity.

#### 4.5.2 Care partner influence and interview context

As with many qualitative studies, the transferability of these findings may be influenced by the specific context and participant characteristics. In some interviews, care partners assisted participants, and especially in remote sessions, it is difficult to establish the extent to which responses reflect the participant versus carer input. This ambiguity should be borne in mind when interpreting error patterns and may warrant specific procedural safeguards and reporting (Carter et al., 2021). The influence of care partner presence on response patterns, while documented in our interviewer observations, warrants systematic investigation in larger samples with sufficient power to detect interaction effects.

#### 4.5.3 Support requirements versus error rates

The apparent divergence between support needs and error rates merits comment. ICECAP-O showed higher error rates yet required the least observed support (4 instances), whereas AQoL-4D had the lowest error rate yet required the most support (14 instances). This suggests that “support” and “error” capture different facets of task demand, and feasibility should be assessed using both metrics. Notably, ICECAP-O was administered in an adapted tabular layout with shortened wording, which may have reduced overt support needs without lowering comprehension errors. Future work could unpack types of support (e.g., reading aloud, rephrasing, example-giving) and test whether specific supports reduce particular error types.

#### 4.5.4 Distinguishing comprehension from construct changes

While we interpreted difficulties with abstract social and emotional concepts (e.g., dignity, valued activities) primarily as comprehension challenges, we cannot rule out that some difficulties may reflect changes in social cognition, apathy, or emotional processing documented in both Alzheimer’s’ and Lewy body dementia. Future research should distinguish between linguistic barriers and changes in the underlying constructs themselves. To broaden beyond verbally mediated assessments, researchers should test complementary approaches, including physiological indicators, behavioural observations, and technology-assisted tools. Moreover, because interviewer observations revealed interactional dynamics within assessment encounters, applying ethnographic methods may illuminate how assessments are co-produced and guide more person-centred outcome assessment in HEOR.

#### 4.5.5 Measure adaptations

ICECAP-SCM and ICECAP-O were presented in adapted tabular formats with abbreviated attribute wording and response options modified to enhance readability and navigation based on Patient and Public Involvement (PPI) feedback (Kinchin et al., 2022). While a standard interviewer script provided full item wording to ensure consistent administration, these adaptations may limit direct comparability with studies using the original formats. The higher error rates we observed for capability measures compared to traditional HRQoL measures should be interpreted in this context.

#### 4.5.6 ICECAP-SCM appropriateness and target population considerations

The ICECAP-SCM is designed for end-of-life contexts, which may help explain our results. Bailey et al. (2016) reported that people in earlier disease stages felt they had to “guess” future experiences when completing ICECAP-SCM items and preferred the ICECAP-A, pointing to a temporal misalignment between current lived experience and hypothetical end-of-life scenarios. This misalignment likely contributed to difficulties among our participants living with mild-to-moderate dementia, particularly given challenges with future-oriented thinking. The higher error rate for participants with Lewy body dementia on ICECAP-SCM (19% vs. 7.8% for AD) likely reflects the measure’s dual cognitive demands: not only does it require future-oriented thinking, but many items (e.g., ‘making important preparations’) tap decision-making and planning capacities that are disproportionately affected by the executive dysfunction characteristic of LBD.

## 5 Conclusion

This study provides the first systematic evidence on the feasibility of self-reporting capability versus health-focused quality of life measures in people living with mild-to-moderate Alzheimer’s disease and Lewy body dementia. Using concurrent think-aloud cognitive interviewing and response process validation, we found that while self-reporting remains feasible across both measure types, capability measures (ICECAP-O, ICECAP-SCM) posed greater cognitive demands than traditional health-related measures (QoL-AD, AQoL-4D), with error rates nearly double (13.7-18.3% vs 5.1-8.4%) despite shorter completion times.

The pattern of errors reveals that the primary barrier to completing quality of life assessments in dementia is not memory retrieval, as commonly assumed, but rather comprehension of abstract concepts and the ability to make evaluative judgements. Capability measures, which require participants to distinguish between what they are able to do (capability) and what they currently do (functioning), proved particularly challenging. This conceptual distinction, already difficult for cognitively healthy adults, becomes substantially more demanding in the context of dementia, where abstract reasoning and future-oriented thinking are compromised.

Importantly, our findings demonstrate that dementia is not a homogeneous condition when it comes to measure feasibility. The distinct cognitive profiles of Alzheimer’s disease and Lewy body dementia produced different error patterns, i.e. participants with Lewy body dementia showed higher comprehension errors and struggled more with abstract, future-oriented content, while those with Alzheimer’s disease showed more response-selection difficulties. These differences highlight the need for measures to be validated and adapted for specific dementia subtypes rather than treating “dementia” as a single category.

For health economics and outcomes research, while capability measures offer theoretical advantages in capturing broader aspects of wellbeing beyond health status, their current formulations may not be sufficiently accessible for routine self-completion by people living with dementia. Traditional health-related measures, grounded in concrete, present-focused questions about functioning, proved more feasible and may be more appropriate for economic evaluations requiring direct self-report. However, this does not diminish the value of capability-oriented constructs such as autonomy, dignity, and valued activities, rather, it highlights the need for alternative measurement approaches that make these constructs more accessible. The path forward may lie in hybrid approaches that anchor capability judgements in recent, concrete experiences, provide clearer definitions and examples for abstract concepts, and consider alternative response formats. Measures that emphasise functioning while capturing the breadth of what people with dementia value may offer a pragmatic balance between conceptual richness and practical feasibility. Importantly, any such developments should involve people living with dementia throughout the design process and recognise that different dementia subtypes and disease stages may require distinct measurement strategies.

Ultimately, this study reinforces the importance of not prematurely shifting to proxy reporting. People living with mild-to-moderate dementia can and should be given the opportunity to report on their own quality of life, provided that measures are designed with their cognitive capabilities in mind. As dementia care increasingly emphasises person-centred approaches, ensuring that the voices of people living with dementia remain central to outcome assessment is not merely a methodological consideration, it is a fundamental requirement for care and policy decisions that reflect their priorities and experiences.

## Supplementary Material

Supplementary materials

## Figures and Tables

**Table 1:** Outcome measures used in the study.

Measure	Conceptual base	Items
ICECAP-SCM adapted*	Capability wellbeing (end-of-life and supportive care)	Wellbeing “at the moment” in terms of choice (being able to have a say), love and affection (being able to be with people who care about you), freedom from physical suffering, freedom from emotional suffering, dignity (being able to maintain dignity and self-respect), support (able to have help and support), and preparation (having the opportunity to make preparations).
ICECAP-O adapted*	Capability wellbeing (older adults)	Person’s capability to have attachment, security, role, enjoyment, and control
QoL-AD	Health-related quality of life (condition specific)	Physical health, energy, mood, living situation, memory, family, marriage, friends, self, ability to do chores, ability to do things for fun, money and life as a whole
AQoL-4D	Health-related quality of life (generic)	Independent living – self-care, household tasks and mobility Relationships - friendships, isolation, and family role Mental health – sleeping, worrying and pain.
		Senses – seeing, hearing and communication

*Note*: ICECAP-SCM and ICECAP-O were presented in a tabular format (see Supplementary materials Tables 1 and 2) with abbreviated attribute wording and response options adapted for this study to enhance readability and navigation. This adaptation was developed in response to Patient and Public Involvement (PPI) feedback (Kinchin et al., 2022), and a standard interviewer script accompanied the table to provide the full item wording and ensure consistent administration.

**Table 2:** Participants characteristics.

Characteristics	*n* (%)
Diagnosis	
Alzheimer’s disease	11 (47.8%)
Lewy Body dementia	12 (52.2%)
Stage	
Mild	12 (52.2%)
Moderate	11 (47.8%)
Gender	
Female	11 (47.8%)
Male	12 (52.2%)
Living in own home/community	23 (100%)
Location*	
Major city	9 (39.1%)
Outside of a major city	7 (30.4%)
Not disclosed	7 (30.4%)
Receive formal carer visits	6 (26.1%)
Care partner present in interview	14 (60.9%)
Age group	
55-59	4 (17.4%)
60-69	5 (21.7%)
70-79	10 (43.5%)
80-85	4 (17.4%)
Symptoms present	
1-5 years	14 (60.94%)
6-10 years	5 (21.7%)
11-19 years	4 (17.4%)
Interview place	
In person	16 (69.6%)
Online video call	7 (30.4%)

Note: Major city includes Dublin, Cork, Limerick, Galway, and Water-ford (defined by population size and economic significance). Outside of a major city refers to smaller towns and rural areas.

**Table 3:** Comparison of completion rates, completion times, support required and error patterns.

	ICECAP-SCM	ICECAP-O	QoL-AD	AQoL-4D
Number of items	7	5	13	12
Average	7.9 (4.4)	6.9 (2.9)	9.2 (5.0)	11.3 (5.0)
completion time				
(min (SD))				
(*n =* 23)				
AD (*n* = 11)	6.6 (3.6)	6.5 (2.5)	8.8 (4.9)	11.0 (3.4)
LBD (*n* = 12)	9.0 (5.0)	7.2 (3.3)	9.6 (5.2)	11.6 (6.2)
Care partner/interviewer	12	4	10	14
support required				
(*n*)*				
Error rate (%)	13.7%	18.3%	8.4%	5.1%
overall (*n* = 23)				
Comprehension	9.9%	10.4%	3.7%	2.6%
Retrieval	0.6%		0.7%	0.4%
Judgement	1.9%	5.2%	2.0%	2.2%
Response	2.3%	2.6%	8.4%	2.6%
*Struggle*	*3.7%*	*1.7%*	*1.3%*	*4.0%*
*Uncertainty*	*3.1%*	*8.7%*	*1.7%*	*3.3%*
Error rate (%) LBD	19.0%	18.3%	9.6%	3.5%
(*n* = 12)				
Comprehension	14.3%	10.0%	5.1%	0.7%
Retrieval	1.2%	-	0.6%	1.7%
Judgement	2.4%	3.3%	3.2%	1.7%
Response	1.2%	5.0%	0.6%	1.4%
*Struggle*	*4.8%*	*3.3%*	*2.6%*	*6.9%*
*Uncertainty*	*4.8%*	*10%*	*1.3%*	*4.2%*
Error rate (%) AD	7.8%	18.2%	7.0%	6.8%
(*n* = 11)				
Comprehension	5.2%	10.9%	2.1%	1.5%
Retrieval			0.8%	
Judgement	1.3%	7.3%	0.8%	3.0%
Response	1.3%	***-***	3.8%	2.3%
*Struggle*	*2.6%*	***-***	*-*	*0.8%*
*Uncertainty*	*1.3%*	*7.3%*	*2.1%*	*2.3%*
Most problematic items	Dignity 9/22 errors, 40.9% of total errors	Doing things that make you feel valued: 13/21 61.9% of total errors	Ability to do things for fun 5/25, 20% of total errors	Relationship with family 3/14, 21% of total errors

*Note*: AD = Alzheimer’s disease; LBD = Lewy body dementia; SD = Standard deviation*Care partner/interviewer support (times) refers to the number of instances during each assessment when the care partner or interviewer provided clarification or influenced the participant’s answer. This includes times when the care partner or interviewer helped interpret questions or guided the participant’s response.

**Table 4:** Types of errors by diagnostic group.

Error type	Description	Total errors across all measures *n* (% of all errors)	Lewy Body dementia (*n* = 12) *n* (% of LBD errors)	Alzheimer’s’ disease (*n* = 11) *n* (% of AD errors)	Example quote
Comprehension	Difficulty understanding question meaning	42 (51.2%)	27 (57.4%)	15 (42.9%)	P8: Um I don’t understand that question, I’m sorry now
Retrieval	Difficulty recalling relevant information	4 (4.9%)	3 (6.4%)	1 (2.9%)	P13: Poor I: Can you tell me more? P13: I don’t have any I: Was it always like that or has it changed?
Judgement	Difficulty making evaluative decisions	20 (24.4%)	10 (21.3%)	10 (28.6%)	On *‘doing things that make you feel valued’,* P14 shifted repeatedly- “I would say... a few,” later “No I’m not able to,” and then, after prompting, “Yes. It probably would be fair to say yes [many]”
Response	Difficulty selecting response option	16 (19.5%)	7 (14.9%)	9 (25.7%)	P10: “I’d say between sometimes and often, sometimes I’ll put it down.” P10: “What do I do here?
Total errors *n *(%)		82 (100%)	47 (57.3%)*	35 (42.7%)*	
Struggle	General difficulty completing question	23	20	3	P10: So am I answering your question?
Uncertainty	Seeking reassurance or expressing doubt	29	18	11	P1: You mean how do I feel in myself or about how I …

Note: Percentages in the “Total errors across all measures” column represent the proportion of each error type out of all 82 errors identified. Percentages in the diagnostic group columns represent the proportion of each error type within that group’s total errors (LBD: 47 errors; AD: 35 errors). The percentages 57.3% and 42.7% in the Total errors row represent the proportion of all errors attributable to each diagnostic group. Struggle and Uncertainty were not classified as errors but captured participant difficulties during completion.

## Data Availability

The authors do not have permission to share data.
