# Supplementary material for: Self-reporting quality of life in mild-to-moderate Alzheimer’s disease and Lewy body dementia: Comparing capability and health-focused measures using response process validation
Source: Soc Sci Med. Author manuscript; Available in PMC 2026 Jun 17. (PMC7619195; doi:10.1016/j.socscimed.2026.119102)
Supplement: Supplementary materials [file EMS212963-supplement-Supplementary_materials.docx]

*Table 1:* Adapted version of ICECAP-SCM used in the study

ABOUT YOUR QUALITY OF LIFE

By placing a tick (X) in **ONE** box in **EACH** group below, please indicate which statement

best describes what you are able to do, and how you are able to be, in your life at the moment.

| **1. Having a say** | Most of the time | Some of the time | A little of the time | Never |
| --- | --- | --- | --- | --- |
| **2. Being with people who**  **care about you** | Most of the time | Some of the time | A little of the time | Never |
| **3. Physical suffering** | Always | Often | Sometimes | Rarely |
| **4. Emotional suffering** | Always | Often | Sometimes | Rarely |
| **5. Dignity** | Most of the time | Some of the time | A little of the time | Never |
| **6. Being supported** | Most of the time | Some of the time | A little of the time | Never |
| **7. Making important preparations for the future** | Most that I want  to make | Some | A few | None |

**Thank you for your help**

*Table 2:* Adapted version of ICECAP-O used in the study

ABOUT YOUR QUALITY OF LIFE

By placing a tick (X) in **ONE** box in **EACH** group below, please indicate which statement

best describes what you are able to do, and how you are able to be, in your life at the moment.

| **1. Love and friendship** | All that I want | A lot | A little | None |
| --- | --- | --- | --- | --- |
| **2. Thinking about the future** | Without any concern | A little concern | Some concern | A lot of concern |
| **3. Doing things that make**  **you feel valued** | All that make me feel  valued | Many | A few | None |
| **4. Enjoyment and pleasure** | All that I want | A lot | A little | None |
| **5. Independence** | Complete | In many things | In a few things | None |

**Thank you for your help**

*Table 3*: Measure-specific performance (overall)

| Measure | Item | Type of error (*n*) |  |  |  | Total errors (*n*) | Struggle | Uncertainty |
| --- | --- | --- | --- | --- | --- | --- | --- | --- |
|  |  | Comprehension | Retrieval | Judgement | Response |  |  |  |
| ICECAP-SCM | Having a say | 3 |  |  | 1 | 4 | 2 |  |
|  | Being with people who care about you | 2 |  |  | 1 | 3 | 1 |  |
|  | Physical suffering |  | 1 |  |  | 1 | 1 | 2 |
|  | Emotional suffering | 2 |  |  |  | 2 |  |  |
|  | Dignity | 6 |  | 3 |  | 9 | 1 | 3 |
|  | Being supported | 2 |  |  |  | 2 | 1 |  |
|  | Making important preparations for the future | 1 |  |  |  | 1 |  |  |
|  | *Total errors (n)* | *16* | *1* | *3* | *2* | *22* | *6* | *5* |
| ICECAP-O | Love and friendship | 1 |  |  | 1 | 2 |  |  |
|  | Thinking about the future | 2 |  |  | 1 | 3 |  | 1 |
|  | Doing things that make you feel valued | 8 |  | 5 |  | 13 | 1 | 8 |
|  | Enjoyment and pleasure |  |  | 1 |  | 1 |  | 1 |
|  | Independence | 1 |  |  | 1 | 1 | 1 |  |
|  | *Total errors (n)* | *12* |  | *6* | *3* | *21* | *2* | *10* |
| QoL-AD | Physical health | 1 |  |  |  | 1 |  |  |
|  | Energy |  |  |  |  | - |  | 1 |
|  | Mood |  |  | 1 |  | 1 |  | 1 |
|  | Living situation |  |  |  | 1 | 1 |  |  |
|  | Memory | 1 |  | 1 | 2 | 4 | 1 |  |
|  | Family |  | 1 |  | 1 | 2 |  |  |
|  | Marriage |  |  |  |  | - |  |  |
|  | Friends |  | 1 |  | 1 | 2 | 1 | 1 |
|  | Self as a whole | 3 |  | 1 |  | 4 |  | 2 |
|  | Ability to do chores around the house | 1 |  | 1 | 1 | 3 |  |  |
|  | Ability to do things for fun | 4 |  | 1 |  | 5 | 2 |  |
|  | Money | 1 |  |  |  | 1 |  |  |
|  | Life as a whole |  |  | 1 |  | 1 |  |  |
|  | *Total errors (n)* | *11* | *2* | *6* | *6* | *25* | *4* | *5* |
| AQoL-4D | Help looking after yourself | 1 |  |  |  | 1 |  | 4 |
|  | Household tasks, such as cooking, cleaning the house, using technology |  |  |  | 2 | 2 |  |  |
|  | Get around home and community |  |  |  |  | - | 1 | 1 |
|  | Relationships with friends, partner, or parents | 1 |  |  |  | 1 | 1 |  |
|  | Relationship with other people |  |  |  |  | - | 1 |  |
|  | Relationship with family | 1 | 1 | 1 |  | 3 | 2 | 1 |
|  | Vision |  |  |  |  | - |  | 1 |
|  | Hearing |  |  | 2 |  | 2 | 2 |  |
|  | When you communicate with others |  |  |  | 1 | 1 | 1 |  |
|  | Sleep |  |  |  | 1 | 1 | 1 |  |
|  | How you generally feel |  |  | 1 | 1 | 2 | 1 | 1 |
|  | Pain or discomfort |  |  | 1 |  | 2 | 1 | 1 |
|  | *Total errors (n)* | *3* | *1* | *5* | *5* | *14* | *11* | *9* |

*Table 4*: Measure-specific performance for participants with LBD

| Measure | Item | Type of error (*n*) |  |  |  | Total errors (*n*) | Struggle | Uncertainty |
| --- | --- | --- | --- | --- | --- | --- | --- | --- |
|  |  | Comprehension | Retrieval | Judgement | Response |  |  |  |
| ICECAP-SCM | Having a say | 3 |  |  | 1 | 4 | 2 |  |
|  | Being with people who care about you | 1 |  |  |  | 1 |  |  |
|  | Physical suffering |  | 1 |  |  | 1 | 1 | 2 |
|  | Emotional suffering | 2 |  |  |  | 2 |  |  |
|  | Dignity | 4 |  | 2 |  | 6 | 1 | 2 |
|  | Being supported | 1 |  |  |  | 1 |  |  |
|  | Making important preparations for the future | 1 |  |  |  | 1 |  |  |
|  | *Total errors (n)* | *12* | *1* | *2* | *1* | *16* | *4* | *4* |
| ICECAP-O | Love and friendship | 1 |  |  | 1 | 2 |  |  |
|  | Thinking about the future | 2 |  |  | 1 | 3 |  | 1 |
|  | Doing things that make you feel valued | 3 |  | 2 |  | 5 | 1 | 5 |
|  | Enjoyment and pleasure |  |  |  |  | - |  |  |
|  | Independence |  |  |  | 1 | 1 | 1 |  |
|  | *Total errors (n)* | *6* |  | *2* | *3* | *11* | *2* | *6* |
| QoL-AD | Physical health | 1 |  |  |  | 1 |  |  |
|  | Energy |  |  |  |  | - |  | 1 |
|  | Mood |  |  |  |  | - |  |  |
|  | Living situation |  |  |  |  | - |  |  |
|  | Memory | 1 |  | 1 |  | 2 | 1 |  |
|  | Family |  | 1 |  |  | 1 |  |  |
|  | Marriage |  |  |  |  | - |  |  |
|  | Friends |  |  |  | 1 | 1 | 1 | 1 |
|  | Self as a whole | 1 |  | 1 |  | 2 |  |  |
|  | Ability to do chores around the house | 1 |  | 1 |  | 2 |  |  |
|  | Ability to do things for fun | 3 |  | 1 |  | 4 | 2 |  |
|  | Money | 1 |  |  |  | 1 |  |  |
|  | Life as a whole |  |  | 1 |  | 1 |  |  |
|  | *Total errors (n)* | *8* | *1* | *5* | *1* | *15* | *4* | *2* |
| AQoL-4D | Help looking after yourself |  |  |  |  |  |  | 4 |
|  | Household tasks |  |  |  | 1 | 1 |  |  |
|  | Get around home and community |  |  |  |  | - | 1 | 1 |
|  | Relationships with friends, partner, or parents | 1 |  |  |  | 1 | 1 |  |
|  | Relationship with other people |  |  |  |  | - | 1 |  |
|  | Relationship with family |  | 1 |  |  | 1 | 2 |  |
|  | Vision |  |  |  |  | - |  | 1 |
|  | Hearing |  |  | 1 |  | 1 | 1 |  |
|  | When you communicate with others |  |  |  | 1 | 1 | 1 |  |
|  | Sleep |  |  |  |  | - | 1 |  |
|  | How you generally feel |  |  |  |  | - | 1 |  |
|  | Pain or discomfort |  |  |  |  | - | 1 |  |
|  | *Total errors (n)* | *1* | *1* | *1* | *2* | *5* | *10* | *6* |

*Table 5:* Measure-specific performance for participants with AD

| Measure | Item | Type of error (n) |  |  |  | Total errors (*n*) | Struggle | Uncertainty |
| --- | --- | --- | --- | --- | --- | --- | --- | --- |
|  |  | Comprehension | Retrieval | Judgement | Response |  |  |  |
| ICECAP-SCM | Having a say |  |  |  |  | - |  |  |
|  | Being with people who care about you | 1 |  |  | 1 | 2 | 1 |  |
|  | Physical suffering |  |  |  |  | - |  |  |
|  | Emotional suffering |  |  |  |  | - |  |  |
|  | Dignity | 2 |  | 1 |  | 3 |  | 1 |
|  | Being supported | 1 |  |  |  | 1 | 1 |  |
|  | Making important preparations for the future |  |  |  |  | - |  |  |
|  | *Total errors (n)* | *4* |  | *1* | *1* | *6* | *2* | *1* |
| ICECAP-O | Love and friendship |  |  |  |  | - |  |  |
|  | Thinking about the future |  |  |  |  | - |  |  |
|  | Doing things that make you feel valued | 5 |  | 3 |  | 8 |  | 3 |
|  | Enjoyment and pleasure |  |  | 1 |  | 1 |  | 1 |
|  | Independence | 1 |  |  |  | 1 |  |  |
|  | *Total errors (n)* | *6* |  | *4* |  | *10* |  | *4* |
| QoL-AD | Physical health |  |  |  |  | - |  |  |
|  | Energy |  |  |  |  | - |  |  |
|  | Mood |  |  | 1 |  | 1 |  | 1 |
|  | Living situation |  |  |  | 1 | 1 |  |  |
|  | Memory |  |  |  | 2 | 2 |  |  |
|  | Family |  |  |  | 1 | 1 |  |  |
|  | Marriage |  |  |  |  | - |  |  |
|  | Friends |  | 1 |  |  | 1 |  |  |
|  | Self as a whole | 2 |  |  |  | 2 |  | 2 |
|  | Ability to do chores around the house |  |  |  | 1 | 1 |  |  |
|  | Ability to do things for fun | 1 |  |  |  | 1 |  |  |
|  | Money |  |  |  |  | - |  |  |
|  | Life as a whole |  |  |  |  | - |  |  |
|  | *Total errors (n)* | *3* | *1* | *1* | *5* | *10* |  | *3* |
| AQoL-4D | Help looking after yourself | 1 |  |  |  | 1 |  |  |
|  | Household tasks |  |  |  | 1 | 1 |  |  |
|  | Get around home and community |  |  |  |  | - |  |  |
|  | Relationships with friends, partner, or parents |  |  |  |  | - |  |  |
|  | Relationship with other people |  |  |  |  | - |  |  |
|  | Relationship with family | 1 |  | 1 |  | 2 |  | 1 |
|  | Vision |  |  |  |  | - |  |  |
|  | Hearing |  |  | 1 |  | 1 | 1 |  |
|  | When you communicate with others |  |  |  |  | - |  |  |
|  | Sleep |  |  |  | 1 | 1 |  |  |
|  | How you generally feel |  |  | 1 | 1 | 2 |  | 1 |
|  | Pain or discomfort |  |  | 1 |  | 1 |  | 1 |
|  | *Total errors (n)* | *2* |  | *4* | *3* | *9* | *1* | *3* |
